# Whole-Genome Sequences of Five *Acinetobacter baumannii* Strains From a Child With Leukemia M2

**DOI:** 10.3389/fmicb.2019.00132

**Published:** 2019-02-06

**Authors:** Jetsi Mancilla-Rojano, Semiramis Castro-Jaimes, Sara A. Ochoa, Miriam Bobadilla del Valle, Victor M. Luna-Pineda, Patricia Bustos, Almudena Laris-González, José Arellano-Galindo, Israel Parra-Ortega, Rigoberto Hernández-Castro, Miguel A. Cevallos, Juan Xicohtencatl-Cortes, Ariadnna Cruz-Córdova

**Affiliations:** ^1^Laboratorio de Investigación en Bacteriología Intestinal, Hospital Infantil de México Federico Gómez, Mexico City, Mexico; ^2^Facultad de Medicina, Universidad Nacional Autónoma de México, Mexico City, Mexico; ^3^Centro de Ciencias Genómicas, Programa de Genómica Evolutiva, Universidad Nacional Autónoma de México, Cuernavaca, Mexico; ^4^Departamento de Enfermedades Infecciosas Instituto Nacional de Ciencias Médicas y de Nutrición “Salvador Zubirán”, Mexico City, Mexico; ^5^Departamento de Epidemiología, Hospital Infantil de México Federico Gómez, Mexico City, Mexico; ^6^Laboratorio de Infectología, Hospital Infantil de México Federico Gómez, Mexico City, Mexico; ^7^Laboratorio Central, Hospital Infantil de México Federico Gómez, Mexico City, Mexico; ^8^Departamento de Ecología de Agentes Patógenos Hospital General “Dr. Manuel Gea González”, Mexico City, Mexico

**Keywords:** *Acinetobacter baumannii*, resistance, molecular typing, adherence, invasion, virulence factors, whole-genome sequence analysis

## Abstract

*Acinetobacter baumannii* is an opportunistic pathogen and is one of the primary etiological agents of healthcare-associated infections (HAIs). *A. baumannii* infections are difficult to treat due to the intrinsic and acquired antibiotic resistance of strains of this bacterium, which frequently limits therapeutic options. In this study, five *A. baumannii* strains (810CP, 433H, 434H, 483H, and A-2), all of which were isolated from a child with leukemia M2, were characterized through antibiotic susceptibility profiling, the detection of genes encoding carbapenem hydrolyzing oxacillinases, pulsed-field gel electrophoresis (PFGE), multilocus sequence typing (MLST), adherence and invasion assays toward the A549 cell line, and the whole-genome sequence (WGS). The five strains showed Multidrug resistant (MDR) profiles and amplification of the *bla*_OXA-23_ gene, belonging to ST758 and grouped into two PFGE clusters. WGS of 810CP revealed the presence of a circular chromosome and two small plasmids, pAba810CPa and pAba810CPb. Both plasmids carried genes encoding the Sp1TA system, although resistance genes were not identified. A gene-by-gene comparison analysis was performed among the *A. baumannii* strains isolated in this study and others *A*. *baumannii* ST758 strains (HIMFG and INCan), showing that 86% of genes were present in all analyzed strains. Interestingly, the 433H, 434H, and 483H strains varied by 8–10 single-nucleotide variants (SNVs), while the A2 and 810CP strains varied by 46 SNVs. Subsequently, an analysis using BacWGSTdb showed that all of our strains had the same resistance genes and were ST758. However, some variations were observed in relation to virulence genes, mainly in the 810CP strain. The genes involved in the synthesis of hepta-acylated lipooligosaccharides, the *pgaABCD* locus encoding poly-β-1-6-*N*-acetylglucosamine, the *ompA* gene, Csu pili, *bap*, the two-component system *bfms*/*bfmR*, a member of the phospholipase D family, and two iron-uptake systems were identified in our *A*. *baumannii* strains genome. The five *A. baumannii* strains isolated from the child were genetically different and showed important characteristics that promote survival in a hospital environment. The elucidation of their genomic sequences provides important information for understanding their epidemiology, antibiotic resistance, and putative virulence factors.

## Introduction

*Acinetobacter baumannii* is an emerging opportunistic pathogen involved in healthcare-associated infections (HAIs) with elevated morbidity and mortality, particularly in immunocompromised patients. *A.* *baumannii* primarily causes ventilator-associated pneumonia and wound and burn infections, but is also an important cause of urinary tract infections and nosocomial septicemia (Gaynes and Edwards, 2005; Dijkshoorn et al., 2007). Treatment for *A. baumannii* infections is complex due to the increasing antibiotic resistance of this pathogen, which involves several intrinsic and acquired resistance mechanisms, such as the production of β-lactamase inhibitors and low-permeability outer membrane and efflux pumps (Peleg et al., 2008). The primary concern regarding HAIs-related *A.* *baumannii* strains is their high resistance to antibiotic therapy and the appearance of new strains that are resistant to all clinically available antibiotics (Peleg et al., 2008).

The study of the molecular epidemiology of bacterial pathogens is an essential tool for establishing control measures for hospital infections, such as the elimination or prevention of the further spread of *A*. *baumannii* strains inside a hospital. Diverse molecular typing methods have been used for epidemiological characterization of HAIs pathogens, including *A*. *baumannii* strains. Pulsed-field gel electrophoresis (PFGE) is a widely used method of choice to discriminate bacterial strains from nosocomial outbreaks (Urwin and Maiden, 2003). Multilocus sequence typing (MLST) is used to study population structures of bacterial pathogens. Several studies have showed typified *A. baumannii* strains using two MLST methods: the Oxford and Pasteur schemes (Bartual et al., 2005; Diancourt et al., 2010).

A study of *A*. *baumannii* of several outbreaks from different countries using the Oxford scheme and PFGE analysis identified a sequence type (ST) with the same subdivision, whereas the Pasteur scheme did not identify differences between outbreaks. Additionally, a major resolution of different outbreaks, including the identification of *gpi* and *gyrB* genes, has been described with the Oxford scheme, although this method is still less discriminative than the PFGE test (Tomaschek et al., 2016). Whole-genome sequencing (WGS) allows putative virulence factors of clinical bacterial strains to be identified (Beres et al., 2010), and in the case of hospital outbreaks, WGS allows colonized patients to be identified and to distinguish the possible transmission route of bacterial populations (Reuter et al., 2013). However, the discrimination criteria for clinical strains from outbreaks and non-outbreaks, as well as between clonal lineages, is not always clearly defined using WGS, and epidemiological data are still required (Leekitcharoenphon et al., 2014). However, the number of SNVs observed between isolates in a temporal frame may bring attention to a putative outbreak (Eyre et al., 2013; Inns et al., 2017). In contrast, the criteria used to establish and discriminate bacteria involved in hospital and nonhospital outbreaks have been well described using PFGE, Rep-polymerase chain reaction (PCR), and MLST tests (Fitzpatrick et al., 2016; Willems et al., 2016).

Comparative genomic analyses of some hypervirulent *A. baumannii* strains have allowed for genomic regions to be identified that contribute to the acquisition of antibiotic resistance, the establishment of colonization and invasion, and ST classification without the MLST analysis requirement (Ou et al., 2015; Zhang et al., 2018). The ST of clinical strains provides relevant information regarding the origin of clonal complexes, including their population distribution, which is epidemiologically important (Higgins et al., 2012). The goal of this study was to compare five *A. baumannii* strains isolated from a child with leukemia M2 using classical molecular typing (PFGE and MLST) and WGS using Illumina and PacBio platforms.

## Materials and Methods

### Identification of *A. baumannii* Strains

The *A. baumannii* strains were cultured on Brucella blood agar from BD Difco (Madrid, Spain) and phenotypically identified at the Laboratorio Clínico Central of HIMFG using a Vitek^®^ 2 automated system (BioMérieux, Marcy l’Étoile, France).

### Antibiotic Susceptibility Tests

Antibiotic susceptibility testing was performed using the broth microdilution method. The antibiotics evaluated included the following: piperacillin (penicillin); piperacillin-tazobactam (β-lactam combination agents); ceftazidime, and ceftriaxone (cephems); imipenem and meropenem (carbapenems); colistin (lipopeptide); gentamicin (aminoglycoside); ciprofloxacin and levofloxacin (fluoroquinolones); trimethoprim-sulfamethoxazole (folate pathway antagonists); and tigecycline (glycylcycline). *A. baumannii* ATCC^®^19606^TM^ was used as a quality control strain, and classification was performed according to the Clinical Laboratory Standards Institute [CLSI], 2018. Susceptibility to tigecycline was interpreted according to the US Food and Drug Administration (FDA) breakpoints for Enterobacteriaceae.

### Detection of *bla*_OXA-LIKE_ Carbapenemase Genes

Genomic DNA was extracted from the *A*. *baumannii* strains using a Quick-DNA Universal kit (Zymo, Irvine, CA, United States), and the *bla*_OXA-LIKE_ genes were amplified by PCR. The specific primers used for PCR are listed in Table 1. PCR assays were performed using the following thermocycling conditions: 94°C for 5 min; 30 cycles at 94°C for 25 s, 52°C for 40 s, and 72°C for 50 s; and a final cycle at 72°C for 6 min. The DNA products were separated by electrophoresis in 1.8% agarose gels and stained with 0.5 mg/mL ethidium bromide solution. The stained gels were visualized and analyzed under a transilluminator (Bio-Rad, San Francisco, CA, United States). *A. baumannii* ATCC^®^19606^TM^ was used as a positive control for *bla*_OXA-51_.

**Table 1:** Specific primers used to amplify *bla*_OXA-LIKE_ genes and MLST data.

Gene	Primer sequences 5’–3’	Amplified (bp)	PCR	Reference
*bla_OXA-23_*	GATCGGATTGGAGAA CAGA	501	OXA	Hujer et al., 2006
	ATTTCTGACCGCATTTCCAT			
*bla_ OXA-24_*	GGTTAGTTGGCCCCCTTAAA	246	OXA	Hujer et al., 2006
	AGTTGACGCAAAAGGGGATT			
*bla_ OXA-51_*	TAATGCTTTGATCGGCCTTG	353	OXA	Hujer et al., 2006
	TGCATTGCACTTCATCTTGG			
*bla_ OXA-58_*	AAGTATTGGGGCTTGTGCTG	453	OXA	Hujer et al., 2006
	CCCCTCTGCGCTCTACATAC			
*gltA* F	AAT TTACAGTGGCACATTAGGTCCC	722	MLST	Bartual et al., 2005
*gltA* R	GCAGAGATACCAGCAGAGATACACG		Amp/Seq	
*gyrB* F	TGAAGG CGGCTTATCTGAGT	594	MLST	Bartual et al., 2005
*gyrB* R	GCTGGGTCTTTTTCCTGACA		Amp/Seq	
*gdhB* 1F	GCTACTTTTATGCAACAGAGCC	774	MLST Amp	Bartual et al., 2005
*gdh* secF	ACCATGCTTTGTTATG		Seq	
*gdhB* 775R	GTTGAGTTGGCGTATGTTGTGC	774	MLST Amp	Bartual et al., 2005
*gdh* secR	GTTGGCGTATGTTGTGC		Seq	
*recA* F	CCTGAATCTTCYGGTAAAAC	425	MLST	Bartual et al., 2005
*recA R*	GTTTCTGGGCTGCCAAACATTAC		Amp/Seq	
*cpn60* F	GGTGCTCAACTTGTTCGTGA	640	MLST	Bartual et al., 2005
*cpn60* R	CACCGAAACCAGGAGCTTTA		Amp/Seq	
*gpi* F	GAAATTTCCGGAGCTCACAA	456	MLST	Bartual et al., 2005
*gpi* R	TCA GGA GCA ATACCCCACTC		Amp/Seq	
*rpoD* F	ACC CGT GAA GGT GAA ATC AG	672	MLST	Bartual et al., 2005
*rpoD* R	TTC AGC TGG AGC TTT AGC AAT		Amp/Seq	


*OXA-23 (*bla_*OXA-23*_*), OXA-24 (*bla_*OXA-24*_*), OXA-51 (*bla_*OXA-51*_*), and OXA-58 (*bla_*OXA-58*_*). Citrate synthase (*gltA*), DNA gyrase subunit B (*gyrB*), glucose dehydrogenase B (*gdhB*), homologous recombination factor (*recA*), 60-kDa chaperonin (*cpn60*), glucose-6 phosphate isomerase (*gpi*); RNA polymerase sigma factor (*rpoD*). Amp, Amplification; Seq, sequencing.*

### PFGE Assays

The *A*. *baumannii* strains were plated onto Brucella blood agar and incubated at 37°C for 18 h. Approximately 5–10 colonies were selected and resuspended in 1 mL of Negative Gram Suspension Buffer (NGSB) (100 mM Tris–HCl and 100 mM EDTA 100, pH 8). From this bacterial suspension, 400 μL was embedded into 1% agarose plugs (SeaKem, Cambrex, Rockland, MD, United States), lysed with 5 mL of lysis buffer at pH 8.0 [0.5 M Tris–HCl, 0.5 M EDTA, 1% *N*-lauryl sarcosine sodium salt, and 25 μL of proteinase K (20 mg/mL)] with constant stirring (200 rpm) and incubated overnight at 54 ± 2°C. Subsequently, the samples were washed (MilliQ water at 50°C and 6× TE buffer) and digested with the enzyme *Apa*I (Promega, Madison, WI, United States). The digested chromosomal DNA was electrophoresed on 1% agarose gels (Bio-Rad, Hercules, CA, United States) using the CHEF MAPPER system (Bio-Rad, Hercules, CA, United States) in 0.5× TBE (AMRESCO, United States) under the following conditions: initial time of 5.0 s, final time of 30.0 s, 6 V/cm, with an inclination angle of 120 and a running time of 24 h. A lambda marker (Biolabs, Hertfordshire, United Kingdom) was used as molecular weight marker. Subsequently, the gels were stained with 0.5 mg/mL of ethidium bromide for 40 min and visualized under UV light. The DNA fragment patterns generated by PFGE were analyzed and compared using NTSYS version 2.2 (Applied Biostatistics, Setauket, New York, NY, United States) with the unweighted pair group method using arithmetic averages (UPGMA) algorithm and the DICE correlation coefficient (Tenover et al., 1995).

### MLST

Amplification of the seven housekeeping genes (OXFORD scheme) was performed according to the protocol proposed by Bartual et al. (2005) using the specific primers listed in Table 1.

Polymerase chain reactions contained 100 ng of genomic DNA from each strain for the five genes (*gltA*, *gyrB*, *recA*, *cpn60*, and *rpoD*) and the following PCR thermocycling conditions were used: 94°C for 2 min; 35 cycles at 94°C for 30 s, 52°C for 30 s, and 72°C for 30 s; and a final cycle of 72°C for 5 min. For the *gdhB* and *gpi* genes, the following PCR thermocycling conditions were used: 94°C for 5 min; 35 cycles at 94°C for 1 min, 57°C for 1 min, and 72°C for 2 min; and a final cycle at 72°C for 7 min. The DNA amplicons were resolved in 1.0% agarose gels in TAE 1× buffer, stained with 0.5 mg/mL ethidium bromide, and visualized under a transilluminator.

Only the *gdhB* and *gpi* genes were cloned. The DNA products were ligated into the cloning vector pJet1.2/blunt (Thermo Fisher Scientific, Waltham, MA, United States). Subsequently, the ligation mixture was used to transform *E. coli* DH5α competent cells, and the resulting colonies carrying the genes cloned into the pJet1.2/blunt vector were verified by PCR assays. The transformed plasmid was extracted using a Plasmid Miniprep kit (Zymo, Irvine, CA, United States), purified, and sequenced by Sanger sequencing. The sequencing reaction was performed using specific primers corresponding to the housekeeping and the cloning vector pJET1.2/blunt using BigDye-Terminator v3.1 Cycle Sequencing and an automatic ABI 3500 Genetic Analyzer (Applied Biosystems, Foster City, CA, United States). The obtained sequences for each gene were analyzed using the database for *A. baumannii* strains^1^ and characterized by a pattern defining its ST.

### Adherence Assays

The type II pneumocyte cell line A549, derived from human lung carcinoma (A549 ATCC^®^CCL85; Manassas, VA, United States), was cultured in Roswell Park Memorial Institute (RPMI) 1640 medium from GIBCO (Thermo Scientific, Waltham, MA, United States) supplemented with 10% fetal bovine serum (FBS) from GIBCO (Thermo Scientific, Waltham, MA, United States). Briefly, cell monolayers at 70–80% confluence in 24-well plates containing 1 mL of RPMI 1640 medium were infected at a MOI of 100:1 and incubated at 37°C with 5% CO_2_ for 4 h. The *A. baumannii* strains used to infect the A549 cells were cultured in Brain Heart Infusion (BHI) broth overnight at 37°C. The nonattached bacteria were removed and washed three times with 1× PBS. Subsequently, the bacteria attached to the cell monolayers were removed by adding 1 mL of 0.1% Triton X-100 (Amresco, Solon, OH, United States), and serial dilutions were plated onto BHI agar plates and incubated to determine the number of colony-forming units (CFU)/mL. The adherence assays were performed in triplicate on three different days, and the data are expressed as the mean of the averages.

### Invasion Assays

The A549 cell monolayers were prepared and infected as previously described in the adherence assay section. The infected cell monolayers were washed with 1× PBS and incubated with RPMI 1640 medium supplemented with 300 μg/mL lysozyme (Sigma–Aldrich, St. Louis, MO, United States) and 300 μg/mL gentamicin (Sigma–Aldrich, St. Louis, MO, United States) for 2 h at 37°C with 5% CO_2_. The infected cell monolayers were washed three times with 1× PBS, detached with 1 mL of 0.1% Triton X-100, and plated onto BHI agar plates. The invasion frequency was calculated as the number of surviving bacteria after treatment with gentamycin and lysozyme divided by the total number of quantified bacteria from the adherence assays (Arikawa and Nishikawa, 2010). The invasion assays were performed in triplicate on three different days, and the data are expressed as the mean of the averages.

### Whole-Genome Sequence Analysis

DNA from the *A. baumannii* strains was obtained using a Puregene Yeast/Bact Kit B from Qiagen following the manufacturer’s instructions. The complete genomic sequence of strain 810CP was generated using reads from one SMRT cell of a PacBio RSII platform. The subreads were assembled *de novo* using the RS hierarchical genome assembly process (HGAP) protocol version 3 in SMRT analysis version 2.3 (Pacific Biosciences). To improve regions of low coverage, a genomic DNA shotgun library was prepared using the standard Illumina TruSeq protocol. Sequencing was performed using Illumina Nextseq500 2 × 75 bp paired-end chemistry, and a hybrid assembly was constructed using Unicycler v0.4.1 (Wick et al., 2017). Contigs corresponding to the chromosome and plasmids in the hybrid assembly were circularized both with Unicycler and a Perl script (available at https://github.com/jfass/apc). Draft genomic sequences of strains 433H, 434H, 483H, and A2 were obtained using Illumina Nextseq500 2 × 75 bp, and the reads were assembled with SPAdes 3.11.0. The sequence statistics are shown in Supplementary Table 1. Functional annotation was performed using the NCBI Prokaryotic Genome Annotation Pipeline and Prokka (Seemann, 2014). Reads were realigned against the final assemblies with bwa-mem (Li and Durbin, 2009), and genomic coverage was calculated using bedtools genomecov (Quinlan, 2014). The acquired antibiotic-resistance genes were identified using ResFinder (Zankari et al., 2012) and the BacWGSTdb platform (Ruan and Feng, 2016). Insertion sequences (ISs) were identified with ISfinder^2^ (Siguier et al., 2006). Prophage-related sequences were identified using PHAST^3^ (Zhou et al., 2011). Genomic islands were predicted using IslandViewer 4 with the SIGI–HMM algorithm, which is part of this computational suit (Waack et al., 2006; Bertelli et al., 2017). Virulence-associated genes were identified using the virulence factor database (VFDB)^4^ (Chen et al., 2005) and the BacWGSTdb platform (Ruan and Feng, 2016). Images of genome comparisons were generated using GenVision, a component of the DNASTAR Lasergene Core Suite. The complete genomes obtained in the present study were compared, and against other Mexican strains belonging to ST758 (Graña-Miraglia et al., 2017) using MUMmer 3.0 (Kurtz et al., 2004) and BLAST (Altschul et al., 1990). Gene content matrices were obtained using Roary (Page et al., 2015), with a minimum 90% identity between coding sequences (CDS) as a requisite for a gene to belong to the same family. Single-nucleotide variants (SNVs) between pairs of strains were assessed using MUMmer from unambiguous mappings. In addition, SNVs from monocopy core genes with no recombination signal detected by PhiPack (Bruen et al., 2006) were extracted using single nucleotide polymorphism (SNP) sites (Page et al., 2015). SNVs counts (omitting indels) and gene content matrices were plotted with ComplexHeatmap (Gu et al., 2016) in R 3.2.2.

### Statistical Analyses

The data were analyzed using IBM SPSS version 19.0 (SPSS Inc., Chicago, IL, United States). Student’s *t*-test was used to compare the adherence and invasion among the clinical strains and ATCC^®^19606^TM^, and *p* ≤ 0.05 was regarded as significant.

### GenBank Accession Number

The GenBank accession numbers of the genome sequences obtained or used in this study are listed in Supplementary Table 2.

## Results

### Clinical Description

The pediatric patient was previously healthy, presented at Hospital Infantil de México Federico Gómez (HIMFG) in December 2014 for pancytopenia and a mediastinal tumor, and by means of bone marrow aspirate, the patient was diagnosed with acute myeloid leukemia subtype M2. Two weeks after receiving the first cycle of chemotherapy, the patient was admitted to the emergency ward for a 24-h evaluation, which was characterized by a fever of 38°C, vomiting, colicky abdominal pain, and decreased stools. The patient did not improve with crystalloid administration and therefore required management with vasoactive drugs. Subsequently, the patient was diagnosed with septic shock, and antimicrobial therapy (meropenem, vancomycin, and amphotericin B) was initiated. The blood cultures and uroculture showed no development of microorganisms.

The pediatric patient entered intensive care for advanced management, persistent high-grade fever, and a systemic inflammatory response with persistence of profound neutropenia. Treatment with voriconazole was supplementary due to suspicion of pulmonary aspergillosis and without improvement in the thermal curve. On the eighth day of hospitalization, the patient presented hemodynamic deterioration with septic shock, which required orotracheal intubation and the reinitiation of vasoactive amines. However, the patient died in less than 24 h due to torpid evolution with septic shock refractory to amines, conditioned acute renal injury, disseminated intravascular coagulation, ventilator deterioration, and multiple organ failure. A total of five strains were obtained from this patient, the first strain was isolated on January 7, 2015 from stool (strain 810CP). Subsequently, three additional strains were isolated on January 11, 2015 from the bloodstream at different times (strains 433H, 434H, and 483H), and a final strain isolated on January 12, 2015 from cerebrospinal fluid (strain A-2) after performing an autopsy.

### *A. baumannii* Strains Were Multidrug Resistant (MDR)

The MIC values for all *A. baumannii* strains exhibited resistance to the following six categories of antibiotics: penicillin, the β-lactam combination, cephem, carbapenems, fluoroquinolones, and the folate pathway antagonists. Only two strains were resistant to gentamycin (433H and A-2). Additionally, the *A. baumannii* strains showed a susceptibility profile to the lipopeptide and glycylcycline categories (Table 2).

**Table 2:** Resistance profiles of *A. baumannii* strains.

Clinical isolate	Antibiotics (MIC μg/mL)										
	PIP	TZP	CAZ	CRO	IPM	MEM	CL	GM	CIP	SXT	TGC
810CP	256	256/4	128	256	64	64	1	8	8	32/608	2
433H	256	256/4	128	256	32	64	1	16	8	32/608	2
434H	256	256/4	128	256	64	64	1	8	8	32/608	2
483H	256	256/4	128	256	64	64	1	8	8	32/608	2
A-2	256	256/4	128	256	32	32	1	16	8	32/608	2
ATCC^®^19606^TM^	32	64/4	32	32	0.25	0.5	0.5	4	1	304/16	2
Cut-off	≥128	≥128/4	≥32	≥64	≥8	≥8	≥4	≥16	≥4	≥4/76	≥8
%R	100	100	100	100	100	100	0	40	100	100	0


*Piperacillin (PIP), piperacillin/tazobactam (TZP), ceftazidime (CAZ), ceftriaxone (CRO), imipenem (IPM), meropenem (MEM), colistin (CL), gentamicin (GM), ciprofloxacin (CIP), trimethoprim/sulfamethoxazole (SXT), tigecycline (TGC), ^∗^Cut-off values for resistance to MIC (μg/mL), percentage of resistant (%R)*.

### *A. baumannii* Strains Harboring *bla*_OXA-51_ and *bla*_OXA-23_ Genes

To determine whether the five *A. baumannii* strains harbored genes related to carbapenem resistance, the presence of *bla*_OXA-LIKE_ genes was assessed by PCR assays. From the five *A. baumannii* strains, a 353-bp product was amplified that corresponded to the *bla*_OXA-51_ gene encoding OXA-51, an intrinsic oxacillinase, and a 501-bp product corresponding to the *bla*_OXA-23_ gene encoding OXA-23, an oxacillinase associated with carbapenem resistance (data not shown). In addition, the genes encoding OXA-24 and OXA-48 were not identified in the five *A. baumannii* strains.

### Clonal Relationship of the *A. baumannii* Strains

**FIGURE 1:**
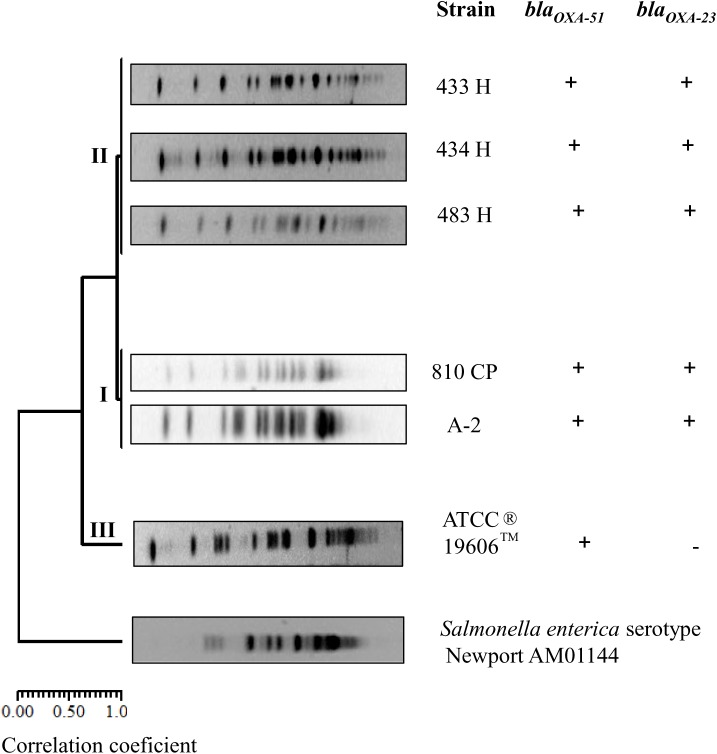
Dendrogram profile displaying the genetic relatedness among the five *A. baumannii* strains based on the PFGE pattern, including the detection of *bla_oxa-51_* and *bla_oxa-23_* genes. *Salmonella enterica* serotype Newport AM01144 was used as external control.

In this study, the *A. baumannii* strains obtained at different times from the same patient were evaluated for their clonal type based on PFGE pattern analyses. The five strains were grouped in two clusters, I and II; while, *A. baumannii* strain ATCC^®^19606^TM^ was grouped as an independent cluster. These clusters displayed a macrorestriction pattern consisting of DNA fragments with molecular weights of 48.5 to 339.5 kb. In cluster I, *A. baumannii* strains A-2 and 810CP showed an identical macrorestriction pattern. In cluster II, *A. baumannii* strains 433H, 434H, and 483H also showed the same macrorestriction pattern, while the *A. baumannii* strain ATCC^®^19606^TM^ was grouped into cluster III. A DICE correlation of 0.9993 and a 95% similarity index was determined in comparisons among the five *A. baumannii* strains. *Salmonella enterica* serotype Newport AM01144 was used as an external control (Figure 1).

### Identification of STs in the *A. baumannii* Strains

**FIGURE 2:**
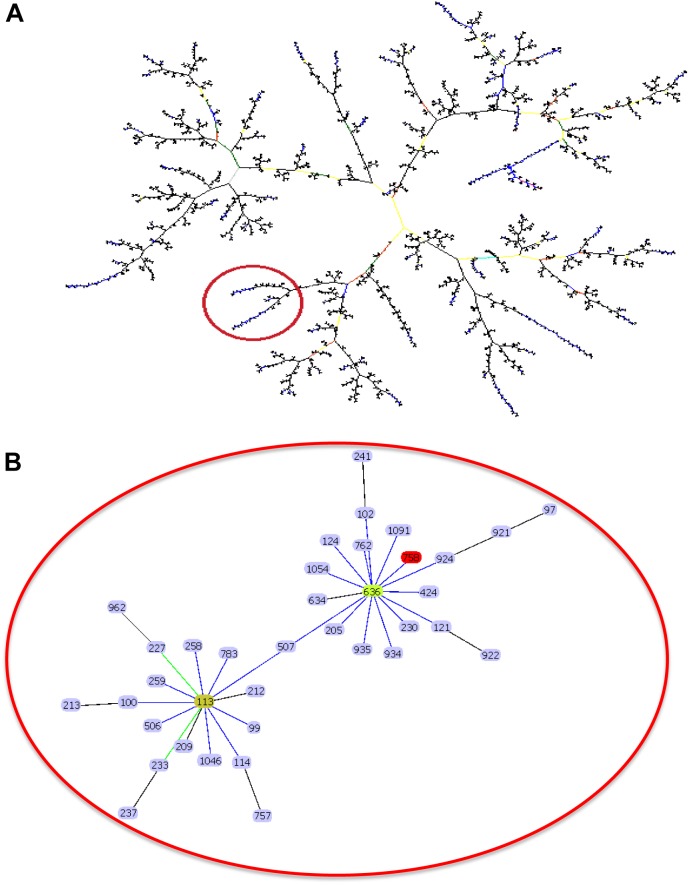
eBURST analysis of *A. baumannii* strains. **(A)** Related and unrelated ST groups to the ST758 in *Acinetobacter baumannii* were compared with 1866 sequences (data generated by MLST) uploaded in the database. **(B)** Zoom showing to ST758 into the CC636.

Multilocus sequence typing sequences of the five *A. baumannii* strains belonged to ST758 with the following allelic profile: *cpn60* (28); *gdhB* (8); *gltA* (1); *gpi* (106); *gyrB* (17); *recA* (10); and *rpoD* (32). According to eBURST, ST758 belongs to clonal complex 636 and shares the same distribution cluster with the other highly frequent STs (Figure 2).

### *A. baumannii* Strains Could Adhere to, but Not Invade, A549 Cells

**FIGURE 3:**
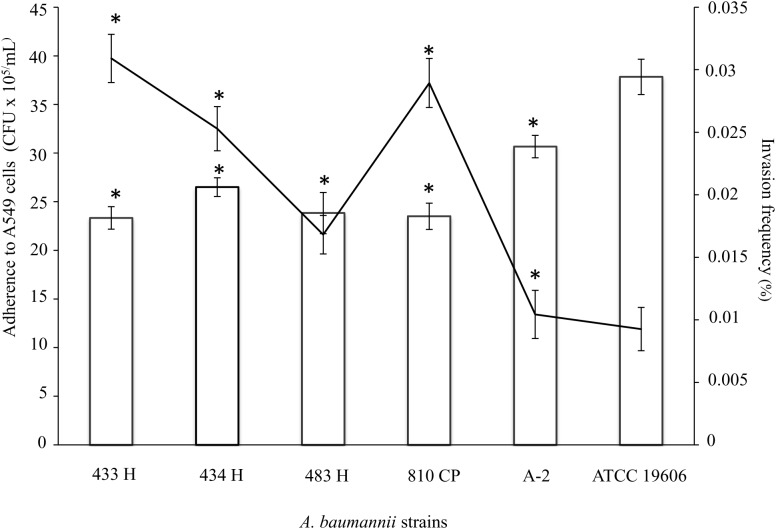
Ability of *A. baumannii* strains to adhere and to invade A549 cells. Quantitative analysis of the adherence of *A. baumannii* strains to A549 cells showed significant increases (*p* = 0.001) after 4 h of infection. Analysis of the invasion frequency showed a significant increase (*p* = 0.028) comparing the 433H strain and other clinical strains, including ATCC^®^19606^TM^. The data (CFU/mL) for the invasion frequency are presented as the means and standard deviations from at least three independent experiments conducted in triplicate. ^∗^Represents a significant difference. The adherence data are presented as bars, and the invasion frequencies are black lines. *A. baumannii* ATCC^®^19606^TM^ was used as a positive control.

The adherence profile of the *A. baumannii* strain ATCC^®^19606^TM^ toward A549 cells after a 4-h infection showed a mean value of 37.8 × 10^6^ CFU/mL, significantly greater (*p* = 0.001) than the adherence profiles of the *A. baumannii* strains isolated in this study, which exhibited mean values between 23.3 × 10^6^ and 30.6 × 10^6^ CFU/mL (Figure 3).

In contrast, the invasion assay using *A*. *baumannii* ATCC^®^19606^TM^ showed a 0.005% invasion frequency, which was significantly lower (*p* = 0.028) than the invasion frequency values of the *A. baumannii* strains isolated in this study (between 0.010 and 0.03%) (Figure 3). However, the *A. baumannii* strains and ATCC^®^19606^TM^ showed lower invasion frequency values than other *A. baumannii* strains.

### Sequencing of the Whole Genome of 810CP

To gain further insights into the genomic structure of *A. baumannii*, the complete genomic sequence of the 810CP strain was obtained using Illumina and PacBio platforms. Our analysis showed that the genome of 810CP consisted of a circular chromosome and two small plasmids (pAba810CPa and pAba810CPb). The general features of this genome are listed in Table 3.

**Table 3:** General features of the genome of *A. baumannii* 810CP.

Features	Chromosome	pAba810CPa	pAba810CPb
Length in bp	4,089,681	5,281	16,095
GC%	39.05	36.77	35.32
Number of protein-coding genes	3,812	8	18
Number of rRNA operons	6	0	0
Number of tRNA/ncRNA/tmRNA genes	67/5/1	0	0
Number of insertion sequences (ISs)	ISAba1 (8)	ISAba27 (1)	0
	ISAba27 (16)		
	ISAba33 (8)		
	ISAba40 (1)		
	ISAba43 (1)		


#### Genomic and Phage-Related Islands

**FIGURE 4:**
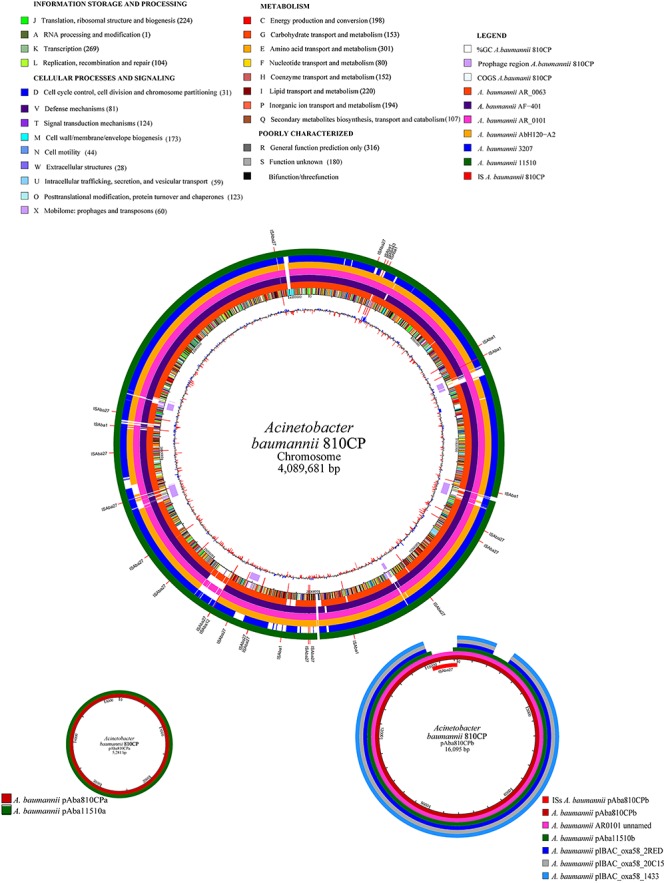
Comparative genome analysis of the *A. baumannii* 810CP strain. The functional classes are represented in different colors. The number of proteins identified for each functional class is shown in parentheses. The *A. baumannii* genome and plasmids that were compared with the *A. baumannii* 810CP strain are shown in different colors.

The 810CP genome possesses a large number of IS elements, especially ISAba27, which is present at 16 chromosomal loci and occurs once in the largest plasmid pAba810CPb (Table 3, Figure 4, and Supplementary Table 3). This genome also contains 11 genomic islands, one of which encodes an essentially hypothetical protein. A 9996-bp resistance island carries genes that confer resistance to aminoglycoside and sulfonamide, as well as other genes encoding three transposases of different families associated with ISL3, IS6, and IS91 (Table 3). Seven regions containing phage-related genes are interspersed throughout the 810CP genome (Figure 4). The largest region is 49498 bp, which was present in only the 810CP strain among the *A. baumannii* genome sequences available in GenBank. Interestingly, these phage-related gene clusters are the most notable differences when comparing the 810CP chromosome sequence with other *A. baumannii* chromosomes sharing at least 90% coverage and 99% nucleotide identity (Figure 4).

#### Antibiotic and Heavy Metal-Resistance Genes

The 810CP genome has a wide range of genes involved in antibiotic and heavy metal resistance. The 810CP chromosome, like most *A. baumannii* strains, carries two genes (*bla*_ACD-25_ and *bla*_OXA-65_) that are related to resistance to β-lactam antibiotics. The *bla*_ACD-25_ gene encodes an AmpC-type β-lactamase (cephalosporinase) that is adjacent to an ISAba1 element. The *bla*_OXA-65_ (*bla*_OXA-51-like_) gene encodes a low-level carbapenamase. In the *A. baumannii* 810CP strain, a *bla*_OXA-239_ gene was also identified, which is an allele belonging to the OXA-23 carbapenem-hydrolyzing class D family and is tightly linked to the ISAba1 element. This strain also contains the *strA* gene, which is involved in streptomycin resistance and associated with another ISAba1 element.

As previously mentioned, the 810CP genome possesses an antibiotic resistance island containing an *aac(6’)-Ia* gene involved in resistance to aminoglycosides and a *sul2* gene linked to sulfonamide resistance. In addition, this strain carries a gene encoding an MdfA efflux pump and has two different chloramphenicol acetyltransferase genes, both of which are linked to a universal stress protein gene. The 810CP strain contains several genes involved in copper resistance that are interspersed throughout its genome. These genes encode proteins that include a TonB-dependent copper receptor (btuB_2), the copper-resistance protein CopB, and the copper-homeostasis protein NlpE.

#### Virulence-Associated Genes

Virulence genes in the 810CP strain were identified using the VFDB collection as queries in a BLASTn search, the results of which are listed in Supplementary Table 4. Briefly, in the 810CP genome, we identified the genes *lpxB*, *lpxD*, *lpxM*, and *lpsB*, which are involved in the synthesis of hepta-acylated lipooligosaccharides (LOS). Genes involved in capsule synthesis were identified within a large cluster in the 810CP chromosome (Supplementary Table 4). Based on the sequencing analysis, we also identified the *pgaABC* locus, encoding proteins involved in the production of poly-β-1-6-*N*-acetylglucosamine, as well as the *ompA* gene, encoding a major component of the outer membrane protein (OmpA), a trimeric porin involved in solute transport and biofilm formation. Other genes with a role in biofilm formation and involved in the synthesis of the Csu pili and the two-component system BfmS were also identified. The 810CP genome also possesses a gene encoding Bap (biofilm-associated protein, locus-tag Aba810CP_04235), a protein with multiple immunoglobulin-like domains localized on the cell surface. Genes encoding the AdeFGH resistance–nodulation–cell division (RND)-type efflux system were identified using the VFDB. In the chromosome of *A. baumannii* 810CP, two genes (locus-tag Aba810CP_02675 and Aba810CP_09700) were identified encoding the phospholipase D family and two types of phospholipase C, one gene (locus_tag Aba810CP_19600) with a match in the VFDB, and a third gene annotated only as “phospholipase.”

In addition, we identified a gene encoding PbpG (locus_tag Aba810CP_18695) and genes involved in the synthesis of two iron-uptake systems mediated by the siderophore acinetobactin.

#### Plasmids

*Acinetobacter baumannii* 810CP possesses two plasmids: pAba810CPa (5281 bp) and pAba810CPb (16095 bp) (Figure 4). The plasmid pAba810CPa harbors only six predicted ORFs, one of which encodes RepB, a protein containing a Rep_3 motif involved in plasmid replication (replicase). This plasmid also possesses genes involved in plasmid mobilization and those related to toxin–antitoxin (TA) systems, although antibiotic-resistance genes were not identified. The pAba810CPb plasmid has 18 predicted ORFs, 8 of which are annotated as hypothetical proteins. Similar to pAba810CPa, pAba810CPb has a TA system (Figure 4). Considering that this plasmid harbors a relaxase-encoding gene (*traA*), it is likely that pAba810CPb is a mobilizable plasmid. pAba810CPb does not encode genes involved in antibiotic resistance, and it contains an integrated ISAba27 element between a TonB-dependent receptor gene and a septicolysin gene. This plasmid also carries genes with a general classification: a CopG family transcriptional regulator, a DNA binding protein, and an acetyltransferase protein. A replicase gene was not identified, indicating that this plasmid has a novel replication system.

### Comparative Genomics of *A. baumannii* ST758 Strains

**FIGURE 5:**
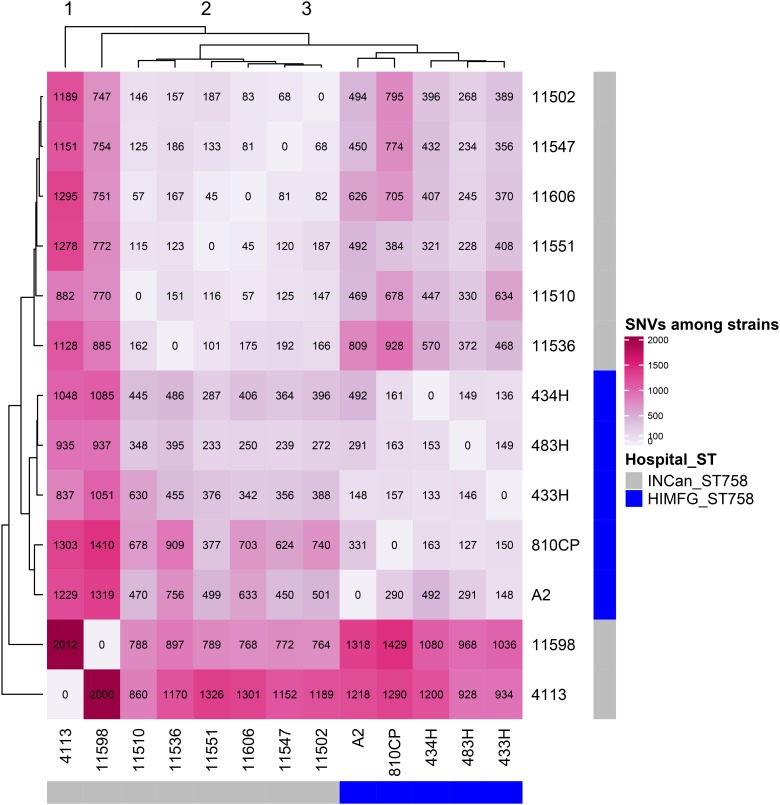
SNVs from raw genomes. Thirteen *A*. *baumannii* ST758 genomes, including five from HIMFG and eight from INCan, were compared. The number of SNVs identified when comparing the raw genomes are shown inside the square. The major variation among strains is shown in pink. HIMFG, Hospital Infantil de Mexico Federico Gómez; INCan, Instituto Nacional de Cancerologia.

**FIGURE 6:**
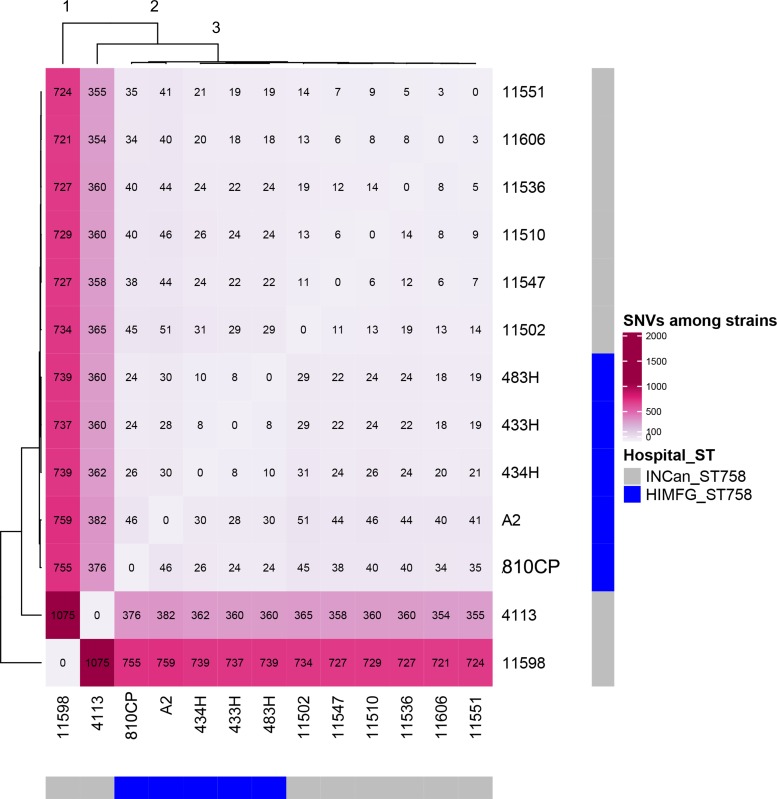
SNVs in core genes with no recombination. Thirteen *A*. *baumannii* ST758 genomes, including five from HIMFG and eight from INCan, were compared. The number of SNVs in core genes with no recombination are shown inside the square. HIMFG, Hospital Infantil de Mexico Federico Gómez; INCan, Instituto Nacional de Cancerologia.

**FIGURE 7:**
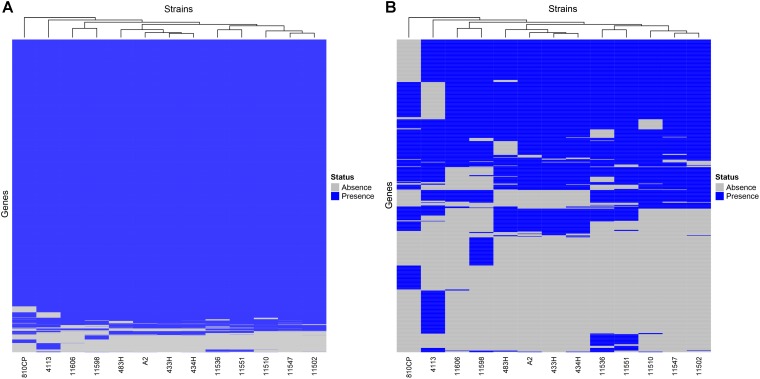
Core and non-core gene heatmaps of *A*. *baumannii* strains. The genomes of 13 ST758 *A*. *baumannii* strains, including 810CP, were compared. **(A)** Core and non-core genes identified in all strains. **(B)** Non-core genes identified in all strains. The genes identified among all strains are shown as follows: the presence or absence of these genes in the genomes are shown in blue and gray, respectively.

The remaining four *A. baumannii* strains (433H, 434H, 483H, and A-2) isolated from same patient were sequenced using only an Illumina platform, and the resulting sequences of these isolates were compared. Analysis of the sequences showed that the strains are closely related but not identical. The five isolates have the same ST (ST758) and possess the same antibiotic resistant genes in the same relative positions. The number of SNVs observed in the complete genomes varied between 127 and 492 (Figure 5), but when only the SNVs from the core genome without recombination signals were calculated, significantly fewer SNVs were identified, ranging from 8 and 30 (Figure 6). Strains 433H, 434H, and 483H varied by 8–10 SNVs, indicating that they are more closely related with each other than with strains A2 and 810CP. However, more dramatic differences were observed in gene content. The five strains have a pangenome of 3811 genes, but their core genome is made up of only 3613 genes. In other words, the non-core genome consists of 198 genes (Figure 7). This observation indicates that rapid gene turnover is crucial for generating genetic diversity in *A. baumannii*, as proposed by Graña-Miraglia et al. (2017). When the genome sequences of other ST758 strains isolated from a different Mexican tertiary care hospital (INCan) were included in the analysis, the variation in SNVs in the complete genomes revealed that the strains 4113 and 11598 showed the greatest variation in the number of SNVs relative to the rest of the assayed strains. It has been proposed that these two strains are hypermutators, and they possess mutations in genes involved in DNA repair (Graña-Miraglia et al., 2017). Interestingly, all the strains isolated from the same patient were grouped together, while the other ST758 strains clustered into the other group (Figure 5, 6). When the gene content of all ST758 strains was analyzed, the number of genes included in the pangenome increased to 4010 genes, while the number of genes in the core genome decreased to 3422 genes (Figure 7). Subsequently, an analysis using BacWGSTdb showed that all of our strains had the same resistance genes and were ST758. However, some variations were observed with respect to virulence genes. Around 80 virulence genes were present in most strains (Supplementary Table 4). However, the strains 810CP and 483H contain only 44 and 77 virulence genes, respectively. Interestingly, the virulence gene data set for the 810CP strain was identical to that of the 11510 strain (an ST758 isolate from INCan).

## Discussion

Acute leukemia is the most common cancer in children younger than 15 years old and has a wide-ranging incidence worldwide, with this disease being particularly prevalent in Hispanic residents in the United States and in Mexican children (Pérez-Saldivar et al., 2011; Metayer et al., 2013). *A. baumannii* is a major cause of morbidity and mortality due to the occurrence of new MDR clinical strains. The risk factors for *A. baumannii* infections, bacteremia, and colonization include severe underlying illness (particularly in critically ill patients and those with hematologic malignancy), prolonged antibiotic therapy with broad spectrum antibiotics, urinary tract infections and catheterization, respiratory tract colonization, infection and endotracheal intubation, and intestinal colonization (Cisneros et al., 1996; Maragakis and Perl, 2008). The overall mortality associated with *A. baumannii* bacteremia ranges between 25 and 54% and depends on the physical condition of the patient. Even in critically ill patients, some studies have shown that the contribution of MDR to *A. baumannii* bacteremia varies between 7.8 and 19%. Septic shock has been reported in up to 42% of patients with bacteremia and crude MDR in patients with bacteremia may reach 70%. However, complicated clinical courses and life-threatening complications are more likely to occur in immunocompromised individuals (Cisneros et al., 1996; Maragakis and Perl, 2008).

Infections associated with carbapenem-resistant *A. baumannii* have been associated with high mortality rates, prolonged hospital stays, and increased health costs (Song et al., 2011; Lee et al., 2014). To nosocomial bacteria such as *A. baumannii* strains, an MDR profile confers a high probability of surviving in a hospital environment and is associated with hospital outbreaks (Cheng et al., 2015; Villalon et al., 2015). Our results characterized the *A. baumannii* strain 810CP as MDR, with the same MIC observed for imipenem and meropenem. Resistance to this antibiotic has increased in recent years worldwide, and in a tertiary care hospital in México, 89.2% of *A. baumannii* strains was observed to be resistant to imipenem (Rosales-Reyes et al., 2017), similar to reports in other countries (Liu et al., 2018).

In the 810CP genome, we identified the AdeFGH RND-efflux system, the overexpression of which confers MDR and the ability to pump antibiotics out of the cell. Furthermore, this system increases biofilm formation together with the overexpression of *adeG*, a component of this system (Coyne et al., 2010). Interestingly, the ISAba1 element identified in the 810CP genome has been previously observed to be associated with the *strA* gene, the encoded product of which confers streptomycin resistance, and it is also involved in promoting ISAba1 overexpression and bacterial susceptibility to these antibiotic classes (Corvec et al., 2003, 2007; Mugnier et al., 2009). The gene encoding the MdfA efflux pump was detected in the 810CP genome, the overexpression of which has been associated with resistance to several antibiotics, such as chloramphenicol and ciprofloxacin (Vila et al., 2007). Other important findings by our group include the identification of a 9996-bp resistance island that has recently been described in other *A. baumannii* strains [e.g., AR0101 (GenBank CP027611.1), 11510 (Graña-Miraglia et al., 2017), AF401 (GenBank NZ_CP018254.1), AbH120-A2 (Merino et al., 2014), and AB030 (Loewen et al., 2014)].

The *bla*_OXA-23_ gene has been considered an important resistance biomarker and is located in either the plasmid or chromosome and is highly prevalent (Mugnier et al., 2010; Opazo et al., 2012; Evans and Amyes, 2014; Luo T.L. et al., 2015). MLST analyses have identified several clonal complexes, groupings of unique genotypes that share at least five loci, and these genotypes have diversified and increased their frequency in the population. For instance, the CC92B clonal complex, which is rarely observed in Latin American isolates but is prevalent in Asia and the United States, is found worldwide and includes carbapenem-resistant clones that harbor the *bla*_OXA-23_ gene encoding OXA-23, representing a risk of propagation of resistant clones (Ji et al., 2013; Wang et al., 2013; Gonzalez-Villoria et al., 2016). Some studies have described genes encoding OXAs that are located in plasmids. However, the *bla*_OXA-23_ gene described by our group was not identified in any of the plasmids studied; therefore, this OXA is encoded in the chromosome (Mugnier et al., 2010). Plasmid pAba810CPa, harboring the gene encoding RepB, contains identical characteristics to plasmid pAba11510a, which was described in another Mexican *A. baumannii* strain belonging to ST758 (Graña-Miraglia et al., 2017). The pAba810CPb plasmid identified in the 810CP strain has a TA system that can enhance the survival of bacterial cells during infection (Díaz-Orejas et al., 2017). Interestingly, genes encoding a TonB-dependent receptor and septicolysin are present in pAba810CPb adjacent to each other. However, many plasmids are not closely related to pAba810CPb, such as pAC12, pAC30a, pAC29a, pAB0057, p2ABYE, pMV01, and pAbaATCC233 (Lean et al., 2016).

In this study, similar PFGE and MLST profiles were obtained for the analyzed *A. baumannii* strains, and the genetic difference of a 388-kb fragment was identified only by PFGE analysis in the strains isolated from the bloodstream compared with the stool and autopsy strains (Tenover et al., 1995). Additionally, five ST758 strains identified by MLST sequencing of *bla*_OXA-23_ (data not shown) and WGS were primarily associated with the OXA-239 allele belonging to the OXA-23 group (Gonzalez-Villoria et al., 2016).

To establish if the five strains were closely related, a comparative genomic analysis was performed. The complete genome sequence of the 810CP strain was obtained using the Illumina and PacBio platforms, while the remaining four *A. baumannii* strains were sequenced only with the Illumina platform. An analysis using BacWGSTdb showed that all of our strains were ST758, as shown by the MLST analysis. Interestingly, ST758 belonging to CC636 corresponds to the Ibero American complex, which has been reported to be the most widespread in Europe, Asia, South Africa, and the United States (Tamayo-Legorreta et al., 2014; Lowings et al., 2015; Vanegas et al., 2015; Gonzalez-Villoria et al., 2016). In Colombia, CC636 has been considered a high-risk clone due to its frequent association with MDR *A*. *baumannii* strains, including carbapenem resistance (Correa et al., 2018).

Comparative genomic analysis was performed among 13 strains isolated from two Mexican hospitals (HIMFG and INCan), all belonging to ST758. The core genome of these strains consists in 34422 genes (86% of the pangenome). The SNVs analysis showed differences among the raw genomes and the genes that are not subject to recombination. Because recombination contributes changes in the genome, is better to use genes that are not subject to recombination to establish relationship among strains. In other bacteria, pairs of sequences varying by 2 SNVs were considered sufficiently closely related to be compatible with a recent direct transmission/acquisition from a common source. Pairs of sequences varying by 0–10 SNVs were considered related through a shared common ancestor sometime during or shortly before the study (∼5 years evolution). Pairs of sequences varying by >10 SNVs were considered genetically distinct (Eyre et al., 2013). Interestingly, the amount of gene content variation in the *A. baumannii* strains was higher than the number of SNVs. These differences observed in our strains can be interpreted in two ways: first, all strains were genetically different, even if they had been isolated from the same patient, and in some cases from the same sample site, suggesting that the patient was infected by a number of distinct, but closely related strains. The other interpretation is that all genetic differences were acquired during the patient illness, indicating, as suggested by Graña-Miraglia et al. (2017), that the A. *baumannii* genome is highly dynamic and that gene turnover may have a crucial role shaping the genome of this microorganism. Additional studies must be performed to clarify this point.

The interface between *A. baumannii* and its environment is the cell envelope and includes the capsule. Genes involved in capsular biosynthesis were identified in our strains. *A. baumannii* cells possess a thick capsular polysaccharide to protect them from different external stresses, including desiccation and host defenses. *A. baumannii* strains lacking the capsule are non-virulent and are affected in biofilm formation (Lees-Miller et al., 2013). Genes involved in the synthesis of hepta-acylated LOS were identified in the genome. *A. baumannii* with LOS mutations are viable, but *in vitro*, they develop growth defects, resulting in severely diminished virulence (Beceiro et al., 2014; Powers and Trent, 2018). The five *A. baumannii* strains and the ATCC^®^19606^TM^ strain with adhesion values to A549 cells ranged from 6.4 × 10^4^ to 4.5 × 10^5^ CFU/mL, similar to values reported in other studies (Eijkelkamp et al., 2011; Giannouli et al., 2013; Na et al., 2016; Ambrosi et al., 2017; Pérez et al., 2017). Sequencing of the whole genomes revealed genes involved in the biogenesis of the type IV pilus, which in Gram-negative bacteria, promotes adherence to human epithelial cells and the formation of microcolonies. However, the role of type IV pili in the *A. baumannii* remains to be investigated (Knutton et al., 1999; Barken et al., 2008; Saldaña-Ahuactzi et al., 2016).

Interestingly, the genes encoding BfmR/BfmS are also present in the genomes. The pili of *A. baumannii* are encoded by the csuA/BABCDE chaperone-usher assembly system, which is controlled by a two-component regulatory system (BfmS and BfmR). This pilus has been associated with twitching-motility and biofilm formation by QS signaling molecules that enhance the expression of the chaperone-usher secretion system (Luo L.M. et al., 2015). The five *A. baumannii* strains obtained from the child with leukemia M2 formed biofilms on polystyrene surfaces when cultured in tryptone soy broth for 24 h at 37°C (data not shown). Biofilm formation and the acquisition of antibiotic-resistance genes in *A. baumannii* are properties that allow this pathogen to survive within a nosocomial environment (Roca et al., 2012). Mutations in the *bfmR*, *bfmS*, and *bap* genes result in decreased or disrupted biofilm formation, while the absence of the *bap* gene decreases adherence to human bronchial cells (Loehfelm et al., 2008; Tomaras et al., 2008; Brossard and Campagnari, 2012). The *pgaABCD* locus in *A. baumannii* encodes poly-β-1-6-*N*-acetylglucosamine, a molecule that plays a role in biofilm formation (Choi et al., 2009). In other pathogens, this molecule plays a major role in cell-to-surface and cell-to-cell adherence and protects cells against host defense mechanisms (Vuong et al., 2004; Sivaranjani et al., 2018). A mutation in the *abaI* gene that encodes the acyl-homoserine lactone autoinducer significantly affects biofilm formation and is favored following the addition of purified acyl-homoserine lactone or overexpression of the *abaI* gene (Bhargava et al., 2010).

*Acinetobacter baumannii* invades epithelial cells via a zipper-like mechanism, which is associated with microfilament and microtubule-dependent uptake mechanisms (Choi et al., 2008). Our data showed that epithelial cells derived from the respiratory tract were more susceptible to *A. baumannii* invasion than non-respiratory tract-derived epithelial cells. However, the strains analyzed in this study showed a low frequency of invasion compared with other invasive pathogens, such as *E. coli*, *Pseudomonas aeruginosa*, *Yersinia enterocolitica*, and *Cronobacter* species (Fleiszig et al., 1995; Huang et al., 1999; Cruz et al., 2011; Uliczka et al., 2011). Recently, OmpA and phosphorylcholine-porin D have been shown to be associated with cell adherence, invasion, and survival within pneumocytes (Mortensen and Skaar, 2012; Ambrosi et al., 2017).

The chromosomes of the assayed *A. baumannii* strains had five genes encoding phospholipases with the ability to hydrolyze phospholipids and contribute to pathogenesis, including the enzymatic activity that occurs during lysis of the host cell membrane (Antunes et al., 2011). These enzymes play a role in the infection and invasion of eukaryotic host cells (Stahl et al., 2015). Phospholipase C has been shown to play a role in virulence in several pathogens, including *A. baumannii*. Recently, phospholipase C in conjunction with elastase has been shown to enhance virulence in an insect model (Kareem et al., 2017).

In the *A. baumannii* genomes, two iron-uptake systems and a gene cluster involved in heme utilization were identified that are important for virulence, as demonstrated in other pathogens (Hom et al., 2013; Ou et al., 2015). Iron is a scarce element in the mammalian host, although it is essential for pathogen survival and infectivity. The systems involved in iron acquisition are very important for virulence. These systems are present in *A. baumannii* genomes and are essential for growth under iron-limiting laboratory conditions (Gaddy et al., 2012). The bioinformatics analysis of the genomes revealed that the TA systems that form a complex in which the antitoxin inhibits toxin activity are encoded by plasmids or chromosomes (Leplae et al., 2011). TA systems have been suggested to mediate bacterial persistence by generating slowly growing cells that are tolerant to antibiotics and environmental changes. Moreover, these systems promote biofilm formation through programmed cell death (Yamaguchi and Inouye, 2009; Fair and Tor, 2014). At least five different TA systems have been identified based on the genomic sequencing of *A. baumannii* strains, including Sp1TA (DUF497/COG3514) (Fernández-García et al., 2016). Among a collection of *A. baumannii* clinical strains from Lithuanian hospitals (88.6% prevalence), HigB/HigAAb and SplTA TA systems were identified as the most abundant. These noncanonical TA systems are most prevalent in clinical *A. baumannii* strains belonging to the ECI and ECII lineages, which are widespread worldwide (Jurenaite et al., 2013). Among five strains, 810CP strain was the most different in relation to virulence genes, this could be associated to this origin, and that it was the first isolated from the child.

In summary, the patient described in this study was severely immunocompromised due to chemotherapy treatment and to the deterioration of health, increasing the risk of developing an infection by *A*. *baumannii*. According to molecular typing results, the strains showed identically PFGE and ST profiles. However, the results of genome sequencing determined that they were different strains with a closely related origin. The *A*. *baumannii* strains described in this study showed important characteristics meriting further investigation, such as with respect to pathway genomics, resistance, and surveillance of molecular epidemiology. The identified ST758 in the *A. baumannii* strains and the associated *bla_OXA-23_* gene are considered genetic biomarkers that contribute to the persistence of these bacteria in the hospital environment. However, further assays are needed to determine whether these strains were endemic to our hospital or had evolved over time. Therefore, genetic studies are required to demonstrate the contribution of putative genes involved in the virulence of *A. baumannii*.

## Ethics Statement

Research Committee (Dr. Juan Garduño Espinosa), Ethics Committee (Dr. Luis Jasso Gutiérrez), and Biosecurity Committee (Dr. Marcela Salazar García) of Hospital Infantil de México Federico Gómez (HIMFG) granted the approval for the development of the protocol HIM/2017/003 SSA.1299. Written informed consent was not required for this study according to the institutional ethical, biosecurity, and investigation Committee due to Central Laboratory from HIMFG provided the *A. baumannii* clinical strains isolates from the child included in this study.

## Author Contributions

AC-C had the initial idea, which was developed into a project together with JX-C and JM-R. JM-R performed the experiments. AC-C, JX-C, MAC, SAO, VML-P, and JA-G analyzed the data. SC-J, MAC, and PB assembled, annotated, and performed bioinformatics analysis of genomes. AL-G reviewed and described the clinical case. MBdV supported the MLST sequencing. AC-C, JX-C, MAC, SAO, JA-G, MBdV, and RH-C contributed reagents and materials. IP-O supplied the *A. baumannii* strains. AC-C, JX-C, and MAC wrote the manuscript, and read and approved the final version. All authors discussed and corrected the manuscript and approved it for publication.

## Conflict of Interest Statement

The authors declare that the research was conducted in the absence of any commercial or financial relationships that could be construed as a potential conflict of interest.

## Footnotes

1 http://pubmlst.org/abaumannii/

2 http://www-is.biotoul.fr

3 http://phast.wishartlab.com

4 http://www.mgc.ac.cn/VFs/main.htm
